# TMPRSS4 induces invasion and proliferation of prostate cancer cells through induction of Slug and cyclin D1

**DOI:** 10.18632/oncotarget.10382

**Published:** 2016-07-02

**Authors:** Yunhee Lee, Dongjoon Ko, Hye-Jin Min, Sol Bi Kim, Hye-Mi Ahn, Younghoon Lee, Semi Kim

**Affiliations:** ^1^ Department of Chemistry, Korea Advanced Institute of Science and Technology, Daejon 34141, Korea; ^2^ Immunotherapy Convergence Research Center, Korea Research Institute of Bioscience and Biotechnology, Daejon 34141, Korea; ^3^ Department of Functional Genomics, Korea University of Science and Technology, Daejon 34113, Korea

**Keywords:** TMPRSS4, prostate cancer, invasion, proliferation, Slug

## Abstract

TMPRSS4 is a novel type II transmembrane serine protease found at the cell surface that is highly expressed in pancreatic, colon, and other cancer tissues. Previously, we demonstrated that TMPRSS4 mediates tumor cell invasion, migration, and metastasis. We also found that TMPRSS4 activates the transcription factor activating protein-1 (AP-1) to induce cancer cell invasion. Here, we explored TMPRSS4-mediated cellular functions and the underlying mechanisms. TMPRSS4 induced Slug, an epithelial-mesenchymal transition (EMT)-inducing transcription factor, and cyclin D1 through activation of AP-1, composed of c-Jun and activating transcription factor (ATF)-2, which resulted in enhanced invasion and proliferation of PC3 prostate cancer cells. In PC3 cells, not only c-Jun but also Slug was required for TMPRSS4-mediated proliferation and invasion. Interestingly, Slug induced phosphorylation of c-Jun and ATF-2 to activate AP-1 through upregulation of Axl, establishing a positive feedback loop between Slug and AP-1, and thus induced cyclin D1, leading to enhanced proliferation. Using data from The Cancer Genome Atlas, we found that Slug expression positively correlated with that of c-Jun and cyclin D1 in human prostate cancers. Expression of Slug was positively correlated with that of cyclin D1 in various cancer cell lines, whereas expression of other EMT-inducing transcription factors was not. This study demonstrates that TMPRSS4 modulates both invasion and proliferation via Slug and cyclin D1, which is a previously unrecognized pathway that may regulate metastasis and cancer progression.

## INTRODUCTION

The metastatic cascade is a complex process consisting of a number of important steps that include local invasion, intravasation, circulation, extravasation, micrometastasis, and metastatic colonization [1]. As an initial step in the metastasis cascade, tumor cells are activated to invade through the epithelial-mesenchymal transition (EMT) process, whereby epithelial cells gradually lose their epithelial features such as cell polarity and cell-cell adhesion and acquire mesenchymal characteristics such as reorganization of the cytoskeleton, enhanced proteolytic activity, and increased motility [2–4]. During EMT, epithelial cells undergo molecular changes; epithelial markers such as E-cadherin are downregulated and mesenchymal markers such as vimentin are upregulated [3]. These changes are usually mediated by EMT-inducing transcription factors directly or indirectly [5–7]. On the other hand, tumor cells undergoing EMT are often growth-arrested because many EMT-inducing transcription factors can directly inhibit proliferation [6]. Therefore, tumor cells may reverse the EMT process to allow metastatic growth in distant sites/organs, although the underlying mechanisms remain unclear.

Slug is a member of the Snail family, whose members are EMT-inducing transcription factors, and is upregulated in metastatic breast cancer, colon cancer, lung cancer, mesothelioma, and melanoma [5]. Slug was recently reported to be involved in metastatic prostate cancer cell invasion and migration [8].

Dysregulation of proteases is a hallmark of tumor progression. Extracellular proteolytic enzymes, such as matrix metalloproteinases and serine proteases, play important roles in cancer cell invasion and metastasis both through direct proteolytic activity and the regulation of cellular signaling and functions [9, 10]. Type II transmembrane proteases (TTSPs) were recently recognized as a new subfamily of serine proteases and all have an extracellular proteolytic domain, a transmembrane domain, and a short cytoplasmic domain [11–13]. Most TTSPs are overexpressed in a variety of tumors in comparison to normal tissues, implicating their functions in tumor development and progression [12]. Recently, a number of studies analyzed the expression of individual TTSPs during tumor progression and investigated the potential roles of these proteases in tumor cell proliferation, migration, and invasion [12].

TMPRSS4, initially referred to as TMPRSS3 [14], is highly overexpressed in various cancers and is associated with poor prognosis in non-small-cell lung cancer, triple-negative breast cancer, cervical cancer, gastric cancer, and colon cancer patients [15]. We previously reported that TMPRSS4 is an important mediator of the migration, invasion, and metastasis of human epithelial cancer cells, and increased TMPRSS4 expression correlates with colorectal cancer stage progression [16–18]. Recently, we demonstrated that TMPRSS4 upregulates expression of the urokinase-type plasminogen activator (uPA) gene, which encodes a well-known serine protease and whose expression correlates with invasion and metastasis, through c-Jun N-terminal kinase (JNK) signaling activation and subsequent activating protein-1 (AP-1) activation to induce cancer cell invasion [19], and that TMPRSS4 upregulates pro-uPA processing through its proteolytic activity [20]. On the other hand, it is not well established whether, and if so, by which mechanisms, TMPRSS4 modulates tumor cell proliferation.

Our previous observation that AP-1 is activated by TMPRSS4 led us to anticipate that TMPRSS4 may modulate both invasion and proliferation. In this study, we found that TMPRSS4 induced AP-1 activation and subsequent expression of Slug and cyclin D1, leading to prostate cancer cell invasion and proliferation. Interestingly, Slug in turn induced AP-1 activity, establishing a positive feedback loop between Slug and AP-1, which led to cyclin D1 expression and cell proliferation. These data provide evidence that the TMPRSS4/AP-1/Slug axis might be exploited as a target for potential anti-cancer therapy.

## RESULTS

### Clinical significance of TMPRSS4 in human prostate cancers

We found that prostate cancer patients with tumors that highly expressed TMPRSS4 (Z > 1.00) had a significantly worse disease-free survival than the remaining patients (Z ≤ 1.00), while prostate cancer patients with tumors that lowly expressed TMPRSS4 (Z < −1.00) had a significantly better disease-free survival than the remaining patients (Z ≥ −1.00) (Supplementary Figure S1) from the analysis of TCGA-generated prostate adenocarcinoma data (MSKCC, Cancer Cell 2010) [21]. Potential roles of TMPRSS4 in tumor cell proliferation and invasion were further explored.

### TMPRSS4 induced proliferation and upregulated slug and cyclin D1

**Figure 1:**
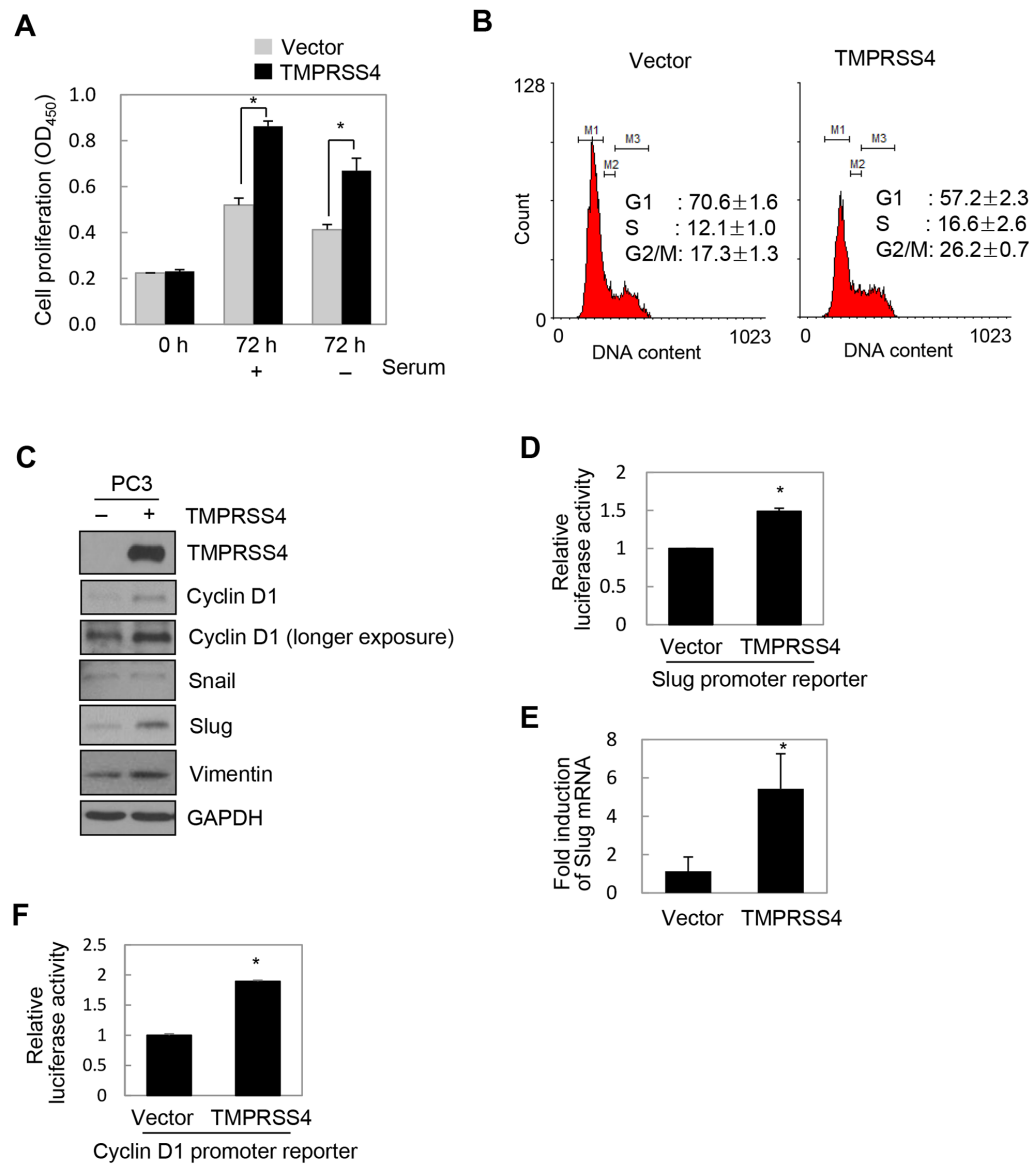
TMPRSS4 induced proliferation and upregulated Slug and cyclin D1 **A, B, C, E.** PC3 cells were transfected with a TMPRSS4 expression vector for 48 h. (A) Transfected cells were seeded into 96-well plates at a density of 3000 cells/well and incubated for 24 h. Cells were further incubated for 48 h in the presence or absence of serum. Cell proliferation was determined by the colorimetric WST assay. (B) Cell cycle analysis of transfected cells using flow cytometry. Percentages of cells in each phase are shown. (C) Transfected cells were lysed and used for immunoblotting. An anti-myc antibody was used to detect myc-tagged TMPRSS4. GAPDH was used as an internal control. **D.** PC3 cells were co-transfected with a TMPRSS4 expression vector and a Slug promoter (−981/+174) reporter construct in the pGL4 vector. Firefly luciferase activity, representing Slug promoter activity, was measured after 48 h and normalized to Renilla luciferase activity to determine the transfection efficiency. (E) Transfected cells were lysed and used for real-time qPCR analysis of Slug mRNA levels. **F.** PC3 cells were co-transfected with a TMPRSS4 expression vector and a cyclin D1 promoter (−962/+134) reporter construct. Luciferase activity was measured as in (D). Values represent mean ± standard deviation (SD). *P < 0.05.

Our previous finding that TMPRSS4 activates AP-1 led us to hypothesize that TMPRSS4 may contribute to the proliferative phenotype of prostate cancer cells. To explore the effect of increased TMPRSS4 expression on cell growth, PC3 prostate cancer cells were transfected with a TMPRSS4 expression vector. TMPRSS4 overexpression significantly increased proliferation of PC3 cells by 66% and 62% in the presence and absence of serum, respectively, over 3 days (Figure 1A). BrdU incorporation analysis revealed that TMPRSS4 significantly enhanced S-phase progression of PC3 cells (Supplementary Figure S2A). Analysis of the cell cycle demonstrated that TMPRSS4-overexpressing PC3 cells displayed a smaller G0/G1-phase population and a larger S-phase population than vector transfectants (Figure 1B). To evaluate the effect of TMPRSS4 overexpression on tumor growth in vivo, TMPRSS4-overexpressing PC3 stable cells were injected subcutaneously into the flank of nude mice. Tumor growth was significantly increased in mouse xenografts with TMPRSS4-overexpressing cells compared with vector transfectants (Supplementary Figure S2B). Immunoblot analysis showed that TMPRSS4 substantially induced cyclin D1 in PC3 cells (Figure 1C). Both Slug and vimentin, but not Snail, were induced by TMPRSS4 in PC3 cells (Figure 1C). To determine whether TMPRSS4 upregulates Slug gene transcription, we transiently co-transfected PC3 cells with the TMPRSS4 expression vector and a reporter plasmid driven by the Slug promoter (−981/+174). TMPRSS4 induced a 1.50-fold increase in Slug promoter activity at 48 h post-transfection (Figure 1D). Real-time quantitative PCR analysis showed that Slug mRNA expression in PC3 cells was significantly induced by TMPRSS4 (Figure 1E). In addition, TMPRSS4 induced a 1.89-fold increase in Cyclin D1 promoter (−962/+134) activity (Figure 1F).

Similarly, TMPRSS4 moderately increased DU145 prostate cancer cell proliferation and moderately induced cyclin D1 expression in these cells (Supplementary Figure S2C, S2D). On the other hand, Snail, but not Slug, was induced by TMPRSS4 in DU145 cells (Supplementary Figure S2D). In addition, TMPRSS4 induced Slug in LNCaP clone FGC and LNCaP-LN3 cells and Snail in only LNCaP-LN3 cells (Supplementary Figure S2E). Other EMT-inducing transcription factors such as Twist1, ZEB1, and ZEB2 were not induced by TMPRSS4 in LNCaP clone FGC, LNCaP-LN3 and PC3 cells (Supplementary Figure S2E). On the other hand, expression of miR-200c, an epithelial marker, was substantially reduced by TMPRS4 overexpression in LNCaP-LN3 and PC3 cells, whereas miR-200c expression was increased by TMPRSS4 overexpression in LNCaP clone FGC cells, suggesting that miR-200c is modulated by TMPRSS4 in a cell context-dependent manner (Supplementary Figure S2F). These observations indicate that TMPRSS4 induced Slug (more frequently) and/or Snail in prostate cancer cells. Together, these results suggest that TMPRSS4 induces prostate cancer cell proliferation through upregulation of cyclin D1.

### JNK signaling activity and c-Jun/ATF-2 were required for TMPRSS4-mediated Slug and cyclin D1 induction

**Figure 2:**
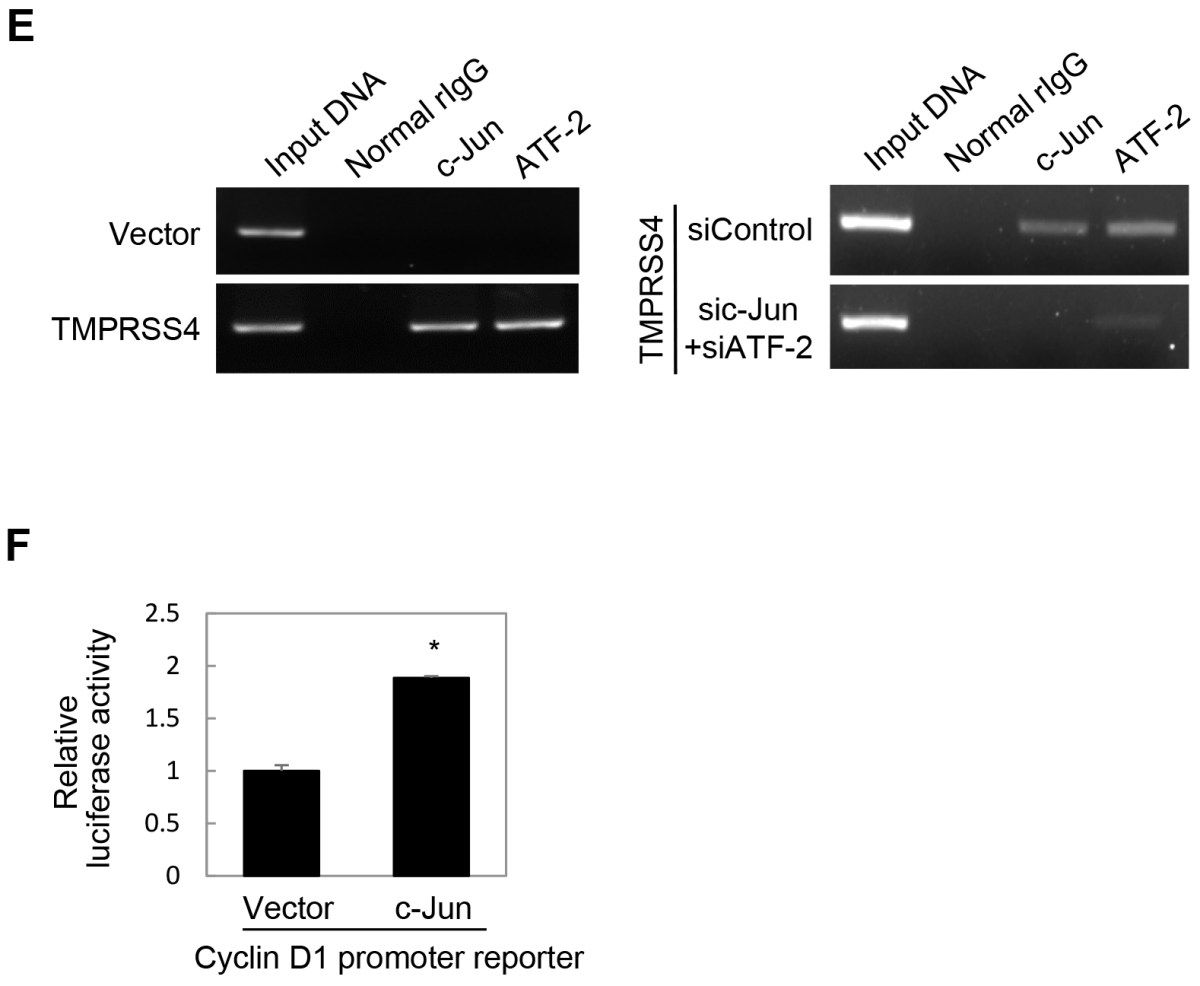
JNK signaling activity and c-Jun/ATF-2 were required for TMPRSS4-mediated Slug and cyclin D1 induction **A.** PC3 cells were transfected with a TMPRSS4 expression vector for 24 h and then treated with pharmacological inhibitors for 24 h before whole-cell lysates were prepared for immunoblotting as described in the Materials and Methods. **B.** PC3 cells were co-transfected with a TMPRSS4 expression vector and an AP-1 reporter plasmid for 48 h. AP-1 activity was determined by a reporter assay as in Figure 1D. **C.** PC3 cells were co-transfected with a TMPRSS4 expression vector or an empty vector and siRNA specific to c-Jun or ATF-2 or negative control siRNA for 48 h. Transfected cells were lysed and used for immunoblotting. An anti-myc antibody was used to detect myc-tagged TMPRSS4. GAPDH was used as an internal control. **D.** Left: PC3 cells were co-transfected with a c-Jun expression vector and a Slug promoter (−981/+174) reporter construct for 48 h. Reporter assays were performed as in Figure 1D. Right: PC3 cells were transfected with a c-Jun expression vector for 48 h and lysed for immunoblotting. **E.** ChIP analysis of the interaction of c-Jun and ATF-2 with the Slug promoter. Left: Chromatin fragments from PC3 cells transfected with a TMPRSS4 expression vector or an empty vector for 48 h were immunoprecipitated with control normal rabbit IgG, anti-c-Jun, or anti-ATF-2 and analyzed via PCR using Slug promoter primers (+1/+172). The input control is in lane 1. Right: ChIP assay using PC3 cells co-transfected with a TMPRSS4 expression vector and siRNAs specific to c-Jun or ATF-2 for 48 h. **F.** PC3 cells were co-transfected with a c-Jun expression vector and a cyclin D1 promoter (−962/+134) reporter construct for 48 h. Reporter assays were performed as in Figure 1D. Values represent mean ± SD. * *P* < 0.05.

We next explored the molecular basis of TMPRSS4-mediated cyclin D1 and Slug induction. We previously observed that TMPRSS4 increases phosphorylation of JNK, ERK1/2, and c-Src in DU145 and PC3 cells [19]. To examine the role of JNK, ERK1/2, and c-Src signaling in TMPRSS4-mediated cyclin D1 and Slug induction, PC3 cells were transiently transfected with the TMPRSS4 expression vector for 24 h and then treated with dimethyl sulfoxide (vehicle), PD098059 (a specific MEK/ERK inhibitor), SP600125 (a specific JNK inhibitor), or SU6656 (a specific c-Src family inhibitor) for 24 h. Inhibition of JNK substantially suppressed phosphorylation of c-Jun and ATF-2 and reduced expression of cyclin D1 and Slug mediated by TMPRSS4, although the JNK inhibitor also moderately reduced phosphorylation of ERK1/2 (Figure 2A). On the other hand, MEK/ERK and c-Src inhibitors moderately suppressed c-Jun and ATF-2 phosphorylation and moderately reduced cyclin D1 and Slug expression (Figure 2A). Consistent with our previous observation in DU145 cells [19], TMPRSS4 significantly activated an AP-1 reporter in PC3 cells (Figure 2B), indicating that TMPRSS4 increased AP-1 transcriptional activity.

Several AP-1 proteins, including c-Jun and ATFs, bind to two AP-1 sites in the cyclin D1 gene regulatory sequences to regulate cyclin D1 transcription [22]. AP-1 (c-Jun/Fra-1) reportedly increases transcription of the Slug gene in breast cancer cells through the AP-1 site in the proximal promoter of the Slug gene [23]. Therefore, we examined whether c-Jun and ATF-2 are required for cyclin D1 and Slug expression induced by TMPRSS4. Immunoblot analysis showed that siRNA-mediated suppression of c-Jun and ATF-2 substantially reduced the induction of cyclin D1 and Slug by TMPRSS4 (Figure 2C). Of note, Slug expression appeared to be more dependent on c-Jun than on ATF-2. To determine whether c-Jun upregulates Slug gene transcription, PC3 cells were co-transfected with the c-Jun expression vector and the Slug promoter (−981/+174) reporter vector containing an AP-1 site (+26/+34). In accordance with the data shown in Figure 2C, c-Jun induced a 1.45-fold increase in Slug promoter activity (Figure 2D). Immunoblot analysis also showed that c-Jun moderately induced Slug expression (Figure 2D). The interaction of c-Jun and ATF-2 with the Slug promoter was examined in PC3 cells transfected with TMPRSS4 using chromatin immunoprecipitation (ChIP). Chromatin fragments containing the Slug promoter (+1/+172) were efficiently pulled down by anti-c-Jun and anti-ATF-2 antibodies from cells transfected with TMPRSS4, whereas chromatin fragments containing the Slug promoter (+1/+172) were not efficiently pulled down from cells transfected with the empty vector (Figure 2E, left). Furthermore, suppression of c-Jun and ATF-2 by siRNA substantially reduced immunoprecipitation of chromatin fragments containing the Slug promoter (+1/+172) in PC3 cells transfected with TMPRSS4 (Figure 2E, right). We also observed that c-Jun induced a 1.89-fold increase in cyclin D1 promoter (−962/+134) activity (Figure 2F). Together, these results indicate that activation of c-Jun and ATF-2 mainly by JNK signaling activity plays an important role in cyclin D1 and Slug expression mediated by TMPRSS4.

### Slug and c-Jun were required for TMPRSS4-mediated invasion and proliferation

**Figure 3:**
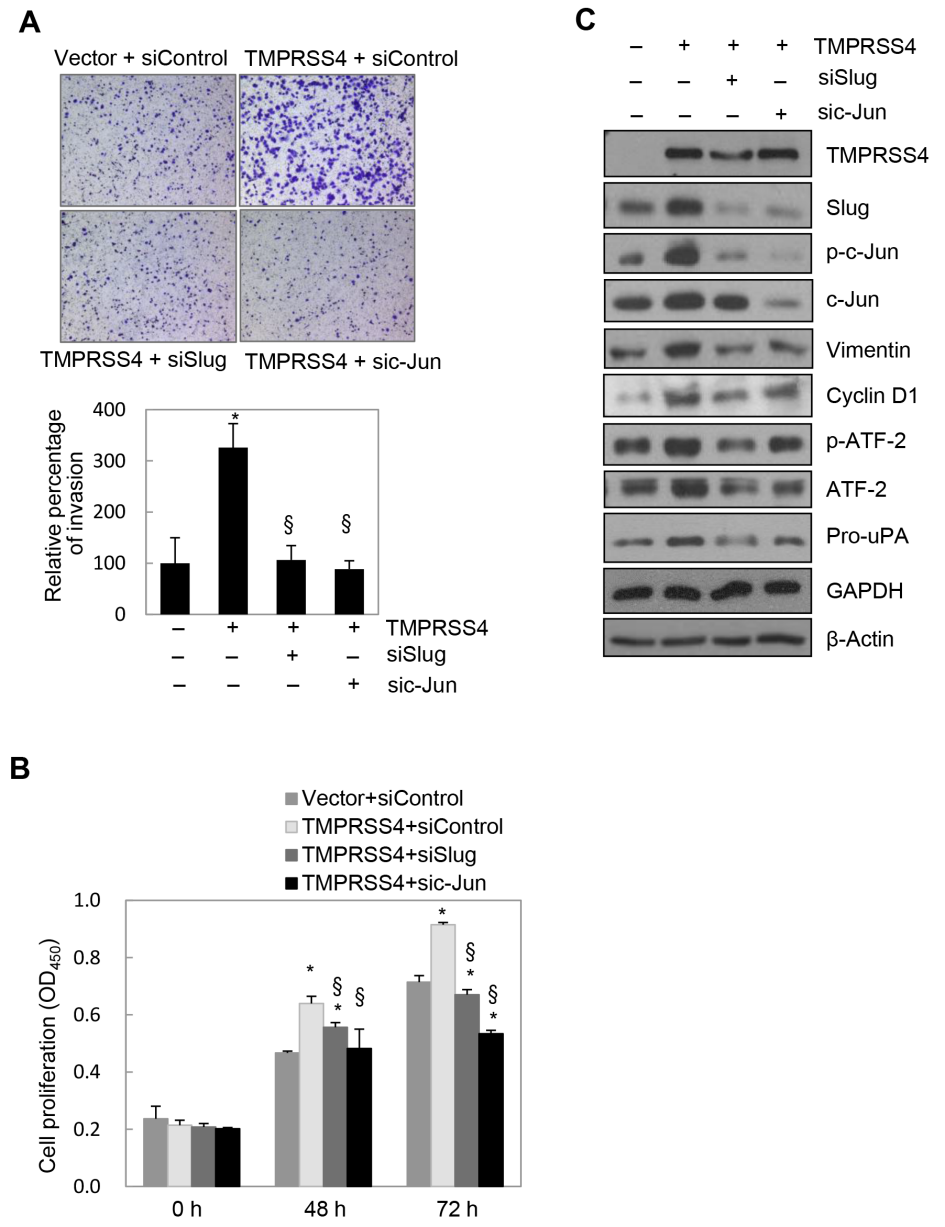
Slug and c-Jun were required for TMPRSS4-mediated invasion and proliferation PC3 cells were co-transfected with a TMPRSS4 expression vector or an empty vector and siRNA specific to Slug or c-Jun or negative control siRNA for 48 h. **A.** Transfected cells were allowed to invade Matrigel (8 × 10^3^ cells) for 48 h. The number of cells that had invaded was counted in five representative (×100) fields per Transwell insert. **B.** Transfected cells were seeded into 96-well plates at a density of 3000 cells/well and incubated for 48 or 72 h. Cell proliferation was determined by the colorimetric WST assay. Values represent mean ± SD. *P < 0.05 compared with empty vector + control siRNA; § *P* < 0.05 compared with TMPRSS4 + control siRNA. **C.** Transfected cells were lysed and used for immunoblotting. An anti-myc antibody was used to detect myc-tagged TMPRSS4. GAPDH was used as an internal control.

We next determined the role of Slug and c-Jun in TMPRSS4-induced invasion and proliferation. PC3 cells were co-transfected with the TMPRSS4 expression vector and siRNA for 48 h prior to invasion and proliferation assays. TMPRSS4-induced invasion was significantly reduced by suppression of Slug or c-Jun (Figure 3A). Of note, elevated invasion mediated by TMPRSS4 may be partially due to moderately increased proliferation in the absence of serum. TMPRSS4-induced proliferation was also significantly decreased by suppression of c-Jun, as expected (Figure 3B). Unexpectedly, depletion of Slug also reduced TMPRSS4-mediated proliferation (Figure 3B).

Immunoblot analysis showed that induction of Slug and cyclin D1 by TMPRSS4 was reduced by knockdown of c-Jun (Figure 3C) (cyclin D1 reduction was moderate), which confirmed the data shown in Figure 2C. c-Jun depletion also reduced the levels of vimentin and pro-uPA (Figure 3C), possibly due to reduced expression of Slug and/or c-Jun itself. c-Jun reportedly interacts with tandem AP-1 sites in the human vimentin promoter region to induce vimentin transcription [24]. Therefore, vimentin expression may be regulated by both c-Jun and Slug. We and other groups previously reported that pro-uPA transcription is activated through AP-1 [19, 25]. Therefore, pro-uPA expression might be regulated by c-Jun and Slug. Phosphorylation of ATF-2 was also reduced by c-Jun knockdown (Figure 3C), possibly due to reduced transcription and/or nuclear transport of ATF-2. Intriguingly, suppression of Slug reduced not only expression of vimentin and pro-uPA mediated by TMPRSS4, but also phosphorylation of c-Jun and ATF-2 and subsequent expression of cyclin D1 induced by TMPRSS4 (Figure 3C). Together, these results suggest that Slug and c-Jun are critical for TMPRSS4-induced invasion and proliferation and indicate the presence of bidirectional (reciprocal) regulation between Slug and c-Jun/AP-1.

### Slug activated AP-1 to induce cyclin D1 expression and cell proliferation

**Figure 4:**
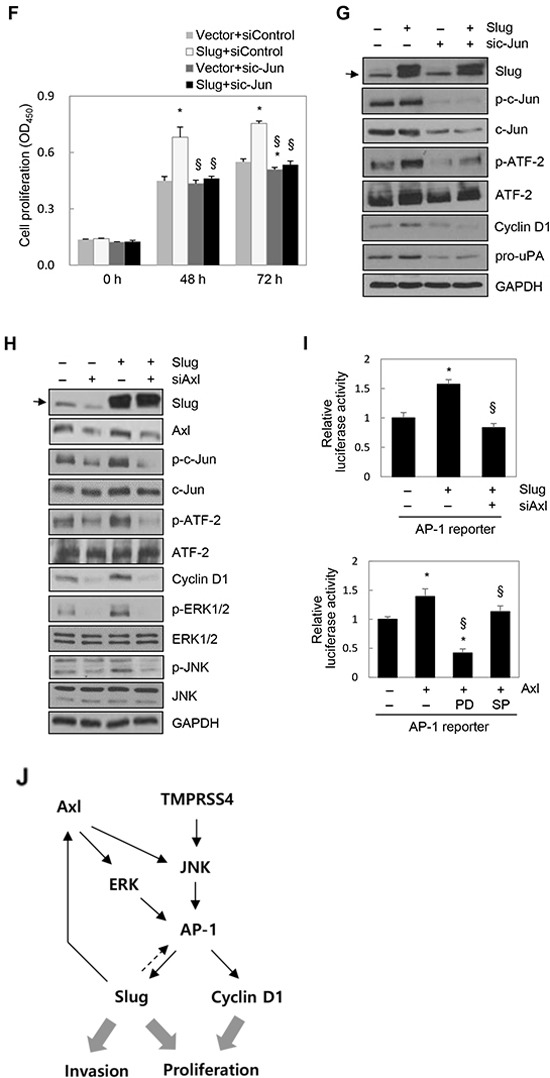
Slug activated AP-1 and induced cyclin D1, leading to cell proliferation **A.** PC3 cells were transfected with a Slug expression vector for 48 h. Transfected cells were lysed and used for immunoblotting. Conditioned medium was collected after an additional 48 h and analyzed by immunoblotting (pro-uPA and active uPA). An anti-myc antibody was used to detect myc-tagged Slug. **B.** Cells were co-transfected with a Slug expression vector and an AP-1 reporter plasmid for 48 h. Reporter assays were performed as in Figure 1D. Values represent mean ± SD. * *P* < 0.05. **C.** HEK293E cells were transfected with a Slug expression vector for 48 h. Transfected cells were lysed and used for immunoblotting. β-Actin was used as an internal control. **D, E.** PC3 cells were transfected with siRNA specific to Slug for 48 h. (D) Transfected cells were seeded into 96-well plates at a density of 3000 cells/well and incubated for 48 or 72 h. Cell proliferation was determined by the colorimetric WST assay. Values represent mean ± SD. * *P* < 0.05. (E) Transfected cells were lysed and used for immunoblotting. **F, G.** PC3 cells were co-transfected with a Slug expression vector or an empty vector and siRNA specific to c-Jun or negative control siRNA for 48 h. (F) Transfected cells were seeded into 96-well plates at a density of 3000 cells/well and incubated for 48 or 72 h. Cell proliferation was determined by the colorimetric WST assay. Values represent mean ± SD. * *P* < 0.05 compared with empty vector + control siRNA; § *P* < 0.05 compared with Slug + control siRNA. (G) Transfected cells were lysed and used for immunoblotting. Arrow indicates endogenous Slug (C, G, H). **H.** PC3 cells were co-transfected with a Slug expression vector and Axl-specific siRNA for 48 h prior to lysis for immunoblot analysis. **I.** Upper: PC3 cells were co-transfected with a Slug expression vector, an AP-1 reporter plasmid, and Axl-specific siRNA for 48 h. Lower: PC3 cells were co-transfected with an Axl expression vector and an AP-1 reporter plasmid for 6 h and then treated with pharmacological inhibitors for 24 h. (Enhanced expression of Axl by the Axl expression vector was confirmed by immunoblotting (Supplementary Figure S3E)). Reporter assays were performed as in Figure 1D. Values represent mean ± SD. * *P* < 0.05 compared with empty vector + control siRNA (upper) or empty vector + DMSO (lower); § *P* < 0.05 compared with Slug + control siRNA (upper) or Axl + DMSO (lower). **J.** A schematic representation of the pathway for TMPRSS4-induced invasion and proliferation in human cancer cells. Axl is involved in Slug-mediated AP-1 activation.

We next investigated whether Slug modulates the activities of c-Jun and ATF-2 and subsequent expression of cyclin D1. PC3 cells were transiently transfected with a Slug expression vector. Immunoblot analysis showed that Slug substantially enhanced phosphorylation of c-Jun and ATF-2 and expression of cyclin D1, vimentin, and pro-uPA in PC3 cells (Figure 4A). In addition, E-cadherin expression was moderately reduced by Slug, as expected (Figure 4A).

To determine whether Slug upregulates AP-1 transcriptional activity, PC3 and HEK293E cells were co-transfected with a Slug expression vector and an AP-1 reporter. Slug increased AP-1 transcriptional activity in PC3 and HEK293E cells by 25% and 62%, respectively (Figure 4B). Immunoblot analysis showed that Slug induced phosphorylation of c-Jun and ATF-2 in HEK293E cells (Figure 4C), confirming the positive correlation between Slug and AP-1 activity. Suppression of endogenous Slug in PC3 cells significantly suppressed proliferation, although this effect was moderate (Figure 4D), and invasion (Supplementary Figure S3A). BrdU incorporation analysis also showed that S-phase progression was moderately reduced by suppression of Slug in PC3 cells (Supplementary Figure S3B). Immunoblot analysis demonstrated that knockdown of Slug reduced phosphorylation of c-Jun and ATF-2 and expression of cyclin D1, pro-uPA, and vimentin (moderately) (Figure 4E). E-cadherin expression was moderately induced by Slug knockdown (Figure 4E). These results suggest that Slug induces cell proliferation, probably through activation of AP-1 and induction of cyclin D1.

We next examined the effects of Slug overexpression on PC3 cell proliferation and the dependency of this process on AP-1. PC3 cells were co-transfected with a Slug expression vector and siRNA specific to c-Jun for 48 h prior to the proliferation assay. We first observed that enhanced expression of Slug significantly increased cell proliferation (Figure 4F). Suppression of c-Jun by siRNA reduced Slug-mediated cell proliferation (Figure 4F). Immunoblot analysis demonstrated that Slug-induced expression of cyclin D1 was reduced following suppression of c-Jun (Figure 4G). Slug-induced pro-uPA expression was also reduced by c-Jun knockdown (Figure 4G). Together, these results suggest that Slug induces cyclin D1 expression and cell proliferation in an AP-1-dependent manner.

To explore the mechanisms underlying Slug-mediated AP-1 activation, we tested the possible involvement of Axl in this process because several previous reports revealed the induction of Axl by Slug [26–28]. PC3 cells were transiently transfected with a Slug expression vector or siRNA specific to Slug for 48 h. Axl expression was induced by Slug at both the mRNA and protein levels, while Axl expression was reduced by suppression of Slug by siRNA at both the mRNA and protein levels (Supplementary Figure S3C), suggesting that Slug upregulates Axl expression in PC3 cells. Analysis of TCGA-generated prostate adenocarcinoma data (MSKCC, Cancer Cell 2010) [21] revealed that Slug expression was significantly correlated with Axl expression (n = 150, r = 0.60, *P* < 0.00001) (Supplementary Figure S3D) when the correlation was analyzed by calculating Pearson's correlation coefficient (r). We then examined whether Axl is required for Slug-mediated c-Jun/ATF-2 phosphorylation and AP-1 activation. PC3 cells were co-transfected with a Slug expression vector and siRNA specific to Axl for 48 h prior to cell lysis. Immunoblot analysis showed that enhanced phosphorylation of c-Jun and ATF-2 mediated by Slug was reduced by knockdown of Axl. Slug-induced expression of cyclin D1 was also reduced following suppression of Axl. Slug-mediated activation of ERK1/2 and JNK, which are downstream signaling molecules of Axl [29, 30], was reduced by knockdown of Axl (Figure 4H). A reporter assay showed that AP-1 activation by Slug was decreased by suppression of Axl (Figure 4I, upper). Furthermore, AP-1 activity was increased by Axl, as expected, and AP-1 activation by Axl was reduced by a JNK inhibitor (SP600125) and a MEK/ERK inhibitor (PD098059) (Figure 4I, lower). These results suggest that Slug enhances c-Jun/ATF-2 phosphorylation and AP-1 activation, at least in part, through upregulation of Axl and subsequent ERK1/2 and JNK signaling activities.

Taken together, these results suggest that TMPRSS4 induces AP-1 activation and subsequent induction of Slug and cyclin D1, leading to prostate cancer cell invasion and proliferation. These results suggest that the TMPRSS4/AP-1/Slug axis contributes to tumor progression (Figure 4J).

### Correlation between slug and cyclin D1 expression in prostate cancers and cancer cell lines

**Figure 5:**
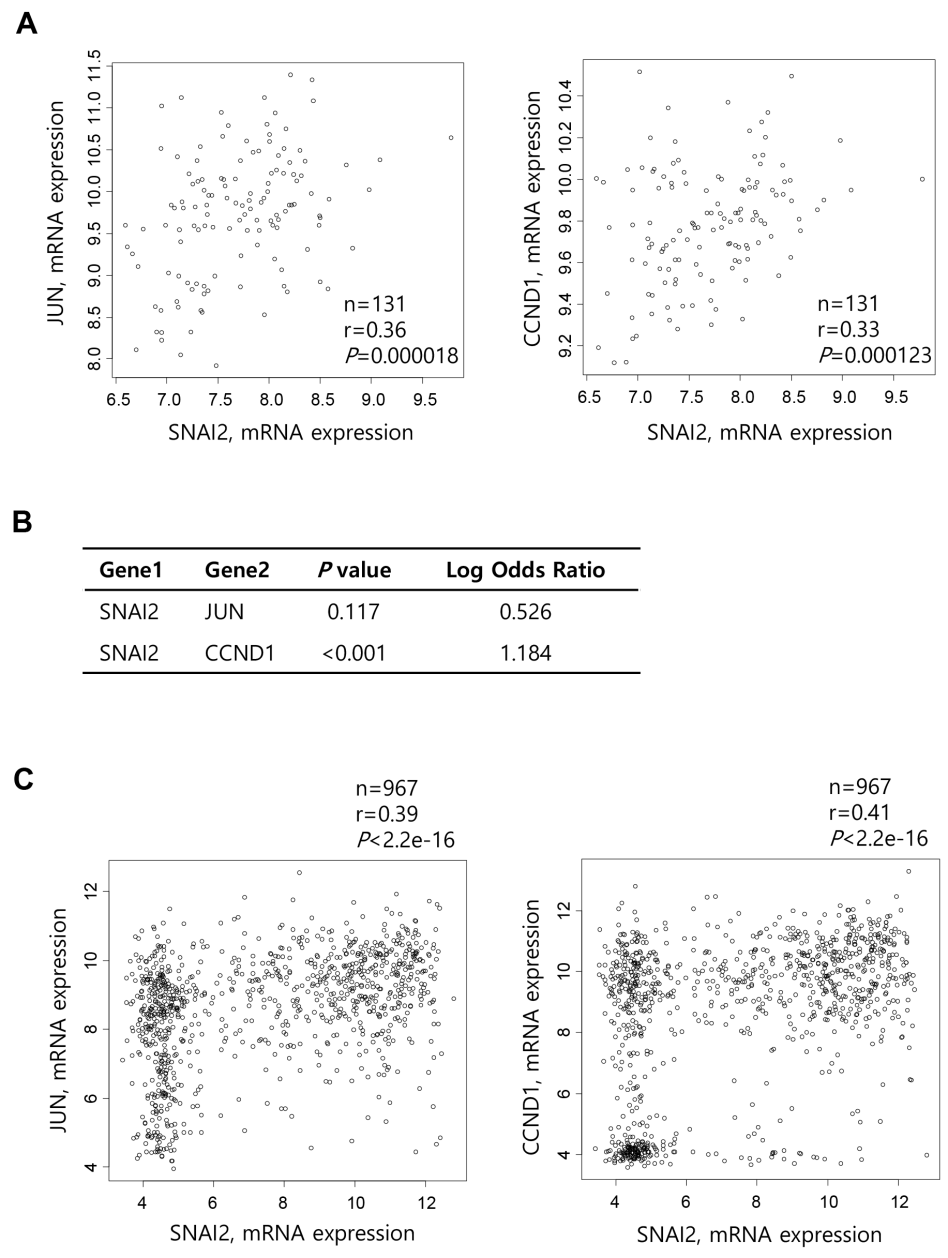
Correlation between Slug and cyclin D1 expression in prostate cancers and various cancer cell lines **A.** Scatter plots examining Slug mRNA expression (x-axis) and c-Jun (left) or cyclin D1 (right) mRNA expression (y-axis) from prostate adenocarcinoma data (MSKCC, Cancer Cell 2010). Correlations were statistically analyzed using the Pearson test. **B.** Fisher's exact test to evaluate the tendency toward the co-occurrence of Slug expression and c-Jun or cyclin D1 expression from data of all prostate tumors with an mRNA expression profile (z-score threshold ± 2.0, n = 150). **C.** Scatter plots examining Slug mRNA expression (x-axis) and c-Jun (left) or cyclin D1 (right) mRNA expression (y-axis) from CCLE data (Novartis/Broad, Nature 2012). Correlations were statistically analyzed using the Pearson test. **D.** Fisher's exact test to evaluate the tendency toward the co-occurrence of cyclin D1 expression and that of Slug or other transcription factors from data of various cancer cell lines (z-score threshold ± 2.0, n = 976).

To determine whether Slug expression correlates with c-Jun or cyclin D1 expression in human cancers, we analyzed TCGA-generated prostate adenocarcinoma data (MSKCC, Cancer Cell 2010) [21]. The correlation was analyzed by calculating Pearson's correlation coefficient (r). Slug expression was significantly correlated with c-Jun (r = 0.36, *P* = 0.000018) and cyclin D1 (r = 0.33, *P* = 0.000123) expression using data from primary tumors (n = 131) (Figure 5A). On the other hand, analysis of tumors (n=150), including metastatic tumors (n = 19), detected a similar positive correlation between expression of Slug and c-Jun (r = 0.39, *P* = 1.016e-06) and a modest positive correlation between expression of Slug and cyclin D1 (r = 0.22, *P* = 0.00595) (Supplementary Figure S4). We also performed Fisher's exact test to evaluate the tendency for the co-occurrence of Slug expression with c-Jun or cyclin D1 expression using all tumors with an mRNA expression profile (n = 150). There was a significant tendency for expression of Slug and cyclin D1 to be positively correlated (*P* < 0.001, Log Odds Ratio = 1.184) (Figure 5B). There was also a tendency for expression of Slug and c-Jun to be positively correlated, although this was insignificant (*P =* 0.117, Log Odds Ratio = 0.526) (Figure 5B).

To further examine this correlation, we interrogated CCLE data (Novartis/Broad, Nature 2012) [31] for the mRNA expression levels of Slug vs. cyclin D1 or c-Jun in various cancer cell lines (n = 967). Slug expression was positively correlated with cyclin D1 and c-Jun expression (r = 0.41, *P* < 2.2e-16, and r = 0.39, *P* < 2.2e-16, respectively; Figure 5C). However, expression of other EMT-inducing transcription factors such as Snail1, Twist1, ZEB1, and ZEB2 was not substantially correlated with expression of cyclin D1 or c-Jun (data not shown). Fisher's exact test showed a significant tendency toward the co-occurrence of cyclin D1 and Slug expression (*P* = 0.003, Log Odds Ratio = 1.169) (Figure 5D). There was also a tendency toward the co-occurrence of Snail and cyclin D1 expression (*P* = 0.014, Log Odds Ratio = 0.603), although this was moderate compared with Slug and cyclin D1 expression. However, Twist1, Twist2, and ZEB1 expression did not show any significant correlation with cyclin D1 expression, while ZEB2 expression was inversely correlated with cyclin D1 expression (Figure 5D).

Suppression of Slug in LNCaP clone FGC, HEK293E, A431, and C8161 cells expressing endogenous Slug reduced cyclin D1 expression (Supplementary Figure S5), confirming a positive correlation between Slug and cyclin D1 expression.

## DISCUSSION

TMPRSS4 is a novel TTSP that is highly expressed in pancreatic, thyroid, lung, colon, and other cancers [15]. We previously reported that TMPRSS4 induces the invasion and EMT of colon cancer cells via upregulation of integrin α5 [16, 17]. We also reported that TMPRSS4 induces invasion of prostate and lung cancer cells through uPA gene expression, which involves AP-1 activation [19]. However, the mechanisms by which TMPRSS4 modulates cancer cell proliferation during tumor progression are not well established. In this study, we report that TMPRSS4 induces Slug and cyclin D1 through AP-1 activation in PC3 prostate cancer cells, leading to both invasion and proliferation. We also found a positive feedback loop (bidirectional upregulation) between Slug and AP-1, which leads to induction of cyclin D1 and cell proliferation, possibly contributing to the accelerated aggressiveness of a malignancy.

On the other hand, it seems that TTSPs other than TMPRSS4 do not induce proliferation. For example, matriptase/MT-SP1 does not affect prostate cancer cell proliferation in vitro [32], although it is required for invasion [32, 33] and induces skin tumorigenesis in a transgenic mouse model [34]. The role of hepsin in cancer growth remains controversial; hepsin is required for hepatoma cell growth [35], whereas it reportedly inhibits the growth of prostate cancer cells [36]. In addition, dipeptidyl peptidase IV, a cell surface protease, induces cell cycle arrest and apoptosis of lung cancer cells [37]. Seprase, a cell surface serine protease that is highly expressed in breast cancer, does not confer a tumor cell growth advantage in vitro [38], although it reportedly promotes growth in vivo [39]. Therefore, our study appears to provide the first evidence that a TTSP family member is positively involved in both proliferation and invasion.

Consistent with our findings, a recent paper reported that TMPRSS4 promotes thyroid cancer proliferation via CREB phosphorylation [40]. It is intriguing that TMPRSS4 induces both invasion (cell migration and extracellular matrix degradation) and proliferation, given that cell proliferation and migration are mutually exclusive processes [41]. Activation of the cell cycle with concomitant inactivation of cell migration may be regarded as a genetically inherited compensation mechanism to maintain cell homeostasis. For example, silencing of cyclin D1 increases the migratory capacity of MDA-MB-231 breast cancer cells [42]. The cyclin D1 network in prostate cancer antagonizes EMT and enhances cancer stem cell populations [43]. Overexpression of ZEB2 in A431 cells results in G_1_ cell cycle arrest through direct transcriptional repression of cyclin D1 and ectopic cyclin D1 uncouples cell cycle arrest from EMT [44], whereas ZEB2 appears to induce proliferation of hippocampal precursors in vivo [45]. Ectopic expression of Snail in MDCK cells decreases proliferation and induces the accumulation of cells in G_1_ phase via repression of cyclin D1 and cyclin D2 and induction of p21(Cip1) [46]. Our study suggests that cancer cells can drastically accelerate the aggressiveness of a malignancy by themselves via TMPRSS4-mediated upregulation of Slug and AP-1 and the simultaneous acquirement of a proliferative and invasive phenotype. It is possible that the positive feedback loop or cooperation between transcription factors plays a critical role in EMT/invasion, the initial step in the metastasis cascade, and additional cellular functions such as proliferation and distant metastatic growth in the metastasis cascade, although this remains to be investigated.

We previously observed that TMPRSS4 is required for NCI-H322 lung cancer cell proliferation, which involves modulation of cell cycle factors [16]. However, we did not observe induction of Slug by TMPRSS4 in NCI-H322 cells (data not shown), although AP-1 is activated by TMPRSS4 [19]. These observations suggest that TMPRSS4 modulates specific signaling pathway(s)/gene expression to induce proliferation in a cell type- or context-dependent manner.

Slug belongs to the Snail family and is a well-known EMT-inducing transcription factor/E-cadherin transcriptional repressor [5]. Slug has been implicated in tumor development and the progression of prostate cancer based on its elevated expression compared with that of other family members [47]. Slug induces specific mesenchymal genes including Slug (autoactivation) [48], ZEB1 [49], and CD147 [50], although it is well known that Slug represses E-cadherin transcription similar to other EMT-inducing transcription factors. We recently observed that Slug induces vimentin [51]. Similarly, Slug induces vimentin and Axl to contribute to the migration and lung metastasis of breast cancer cells [27]. uPA is reportedly induced by ZEB1 through E-box sites in the uPA gene promoter [52]. Therefore, Slug may directly contribute to transcriptional activation of uPA and vimentin.

On the other hand, in regard to proliferation, Slug reportedly downregulates cyclin D1 expression to inhibit the proliferation of PC3 and DU145 prostate cancer cells [47]. By contrast, it is also reported that Slug is required for the proliferation and invasion of PC3 prostate cancer cells [8]. Thus, the role of Slug in the regulation of prostate cancer cell proliferation remains unclear. At present, we cannot completely explain the discrepancy between these results, including those presented in the present study.

Clinically, we found several associations involving Slug expression that are consistent with our experimental data. Analysis of prostate cancer from TCGA data demonstrated a significant positive correlation between expression of Slug and that of cyclin D1 and c-Jun. Our analysis of various cancer cell lines from CCLE data demonstrated that expression of Slug was significantly positively correlated with that of cyclin D1, whereas expression of other EMT-inducing transcription factors was not. Therefore, Slug may play a unique role in concomitant induction of cell proliferation and invasion/EMT, although the precise molecular basis for this needs to be investigated. Activation of c-Jun and ATF-2 by Slug may be an important pathway, although we cannot completely rule out the possibility that Slug induces other AP-1 dimers or other transcription factors that modulate cell cycle factors.

In our attempt to explore how Slug enhances phosphorylation of c-Jun and ATF-2 and subsequent transcriptional activity of AP-1, we demonstrated that Axl plays a role in this process. Consistently, several groups reported induction of Axl by Slug. For example, Axl is upregulated by Slug and Snail in MCF10A immortalized breast epithelial cells and plays a critical role in breast cancer cell invasion [26], and Slug induces the expression of Axl among 49 receptor tyrosine kinases at the transcriptional level to activate Axl, which maintains Slug expression through a positive feedback loop [28]. In addition, the Gas6/Axl pathway upregulates Slug expression through MAPK [29, 51], suggesting a positive feedback loop between Slug and Axl during cancer progression. On the other hand, we previously reported that ZEB2 directly interacts/cooperates with Sp1 to function as a transcriptional activator of mesenchymal genes (vimentin, integrin α5, and cadherin-11) to induce invasion [53]. We also found that functional cooperation between Twist1 and AP-1 induces EMT and invasion [54].

In conclusion, these data provide an important insight into the molecular basis of TMPRSS4-mediated cellular functions. The TMPRSS4/AP-1/Slug axis might be exploited as a target for potential anti-cancer therapy, possibly targeting both cell proliferation and migration.

## MATERIALS AND METHODS

### Cell lines

PC3, DU145, LNCaP clone FGC (prostate cancer), and A431 (skin cancer) cell lines were purchased from the American Type Culture Collection (ATCC), Manassas, VA, USA and were maintained in RPMI1640 containing 10% fetal bovine serum at 37°C/5% CO_2_. LNCaP-LN3 (prostate cancer) and SNU-398 (liver cancer) cell lines were purchased from the Korean Cell Line Bank (KCLB), Seoul, Korea and were maintained in RPMI1640 containing 10% fetal bovine serum. Human embryonic kidney 293E (HEK293E) cells were purchased from ATCC and were maintained in DMEM containing 10% fetal bovine serum. C8161 cells (melanoma) were a kind gift from Dr. C-R Jung (KRIBB, Korea) [55].

### Transfection with expression vectors and small interfering RNA (siRNA)

The TMPRSS4-expressing construct pCMV-myc-TMPRSS4 was described previously [16]. The c-Jun-expressing vector was a kind gift from Dr. Eitan Shaulian (Hebrew University Medical School, Israel) [56]. The Myc-tagged Slug vector was kindly provided by Addgene (#31698; Cambridge, MA, USA). The Axl-expressing vector (pCDNA AXL myc-his) was a kind gift from Dr. Rosa Marina Melillo (DBPCM/IEOS, Italy) [57]. Cells were transfected using Lipofectamine 2000 (Invitrogen, Carlsbad, CA, USA). At 48 h after transfection, cells were lysed or the medium was changed to serum-free medium. Conditioned medium was collected at 48 h. Cells were transfected with siRNA specific to either c-Jun (5′-GAUGGAAACGACCUUCUAUTT-3′), ATF-2 (5′-AAUGAAGUGGCACAGCUGATT-3′), Slug (5′-GAGGAAAGACTACAGTCCAAGTT-3′) or Axl (Santa Cruz Biotechnology, Santa Cruz, CA, USA) using Lipofectamine 2000 for 48 h. In certain experiments, cells were co-transfected with siRNA and a plasmid for 48 h.

### Immunoblot analysis

Whole-cell lysates were prepared using RIPA buffer as described previously [19] and analyzed using the following primary antibodies: anti-uPA, anti-c-Src, anti-ZEB1, anti-β-actin, and anti-GAPDH (Santa Cruz Biotechnology); anti-vimentin (Sigma, St Louis, MO, USA); anti-myc (Upstate Biotechnology, Lake Placid, NY, USA); anti-Slug, anti-Snail, anti-cyclin D1, anti-phospho-c-Jun(S63), anti-c-Jun, anti-phospho-ATF-2(T71), anti-phospho-extracellular signal-regulated kinase 1/2 (ERK1/2), anti-ERK1/2, anti-phospho-c-Src(Y416), anti-phospho-JNK(T183/Y185), and anti-JNK (Cell Signaling Tech., Danvers, MA, USA); anti-Twist1 (Abcam, Cambridge, MA, USA); anti-ZEB2 (Active Motif, Tokyo, Japan); anti-Axl (R&D systems, Minneapolis, MN, USA); anti-TMPRSS4 (in-house) [19]. Where indicated, cells were transiently transfected for 24 h and then treated with 40 mM PD98059, 15 mM SP600125, 3 mM SU6656 (Sigma), or 0.4% dimethyl sulfoxide (DMSO) for 24 h before lysate preparation.

### Cell proliferation and BrdU incorporation assays

Cell proliferation was determined by the colorimetric WST assay (Takara Bio Inc., Otsu, Shiga, Japan). Briefly, cells transfected with siRNA for 48 h were seeded into 96-well plates at a density of 3000 cells/well and incubated for 24 h in the presence of serum. Thereafter, cells were further incubated for 24 or 48 h in the presence or absence of serum. Cells were then incubated with WST reagent (one-tenth of the medium volume) and formazan dye formation was determined by measuring absorbance at 450 nm using a spectrophotometric microplate reader (Molecular Devices, Sunnyvale, CA, USA).

5-Bromo-2′-Deoxyuridine (BrdU) incorporation analysis to measure DNA synthesis was performed using a Cell proliferation ELISA, BrdU (colorimetric) kit (Roche, Manheim, Germany) according to manufacturer's instructions. Briefly, cells transfected for 48 h were seeded into 96-well plates and incubated for 48 h. Then, the cells were incubated with 10 mM BrdU for 2 or 6 h before fixation and DNA denaturation. Cells were incubated with peroxidase-conjugated antibody against BrdU. Color was developed with tetramethyl-benzidine (TMB) substrate and analyzed by measuring absorbance at 370 nm (reference wavelength: 492 nm).

### Cell cycle analysis by flow cytometry

PC3 cells transfected with a TMPRSS4 expression vector or an empty vector for 48 h were harvested, washed with PBS, and fixed in 75% ethanol on ice for 30 min. The fixed cells were stained with 100 μg/ml propidium iodide solution containing 0.3 mg/ml RNase A and 0.2% BSA for 15 min at 25°C. Cells were then analyzed for relative DNA content using a FACSCalibur (BD Immunocytometry System, San Jose, CA).

### Reverse transcription-PCR (RT-PCR)

Total RNA was isolated using TRIzol (Invitrogen), and cDNA was synthesized using reverse transcriptase (Bioneer, Daejon, Korea). Real-time quantitative PCR was performed using SYBR Green (PKT, Seoul, Korea) on a Rotor-Gene 6000 real-time rotary analyzer (Corbett, San Francisco, CA, USA) with Slug-specific primers (5′-ATACCACAACCAGAGATCCTCA-3′ and 5′-GACTCACTCGCCCCAAAGATG-3′) and GAPDH-specific primers (5′-CATGACCACAGTCCATGCCAT-3′ and 5′-AAGGCCATGCCAGTGAGCTTC-3′) with an annealing temperature of 61°C. Semi-quantitative PCR amplification was performed with Axl-specific primers (5′-AAGCGGTCT GCATGAAGGAA-3′ and 5′-TTGACTGGCATCTTGGCGAT-3′) and GAPDH-specific primers (5′-TGATGACATCAAGAAGGTGGTGAAG-3′ and 5′-TCCTTGGAGGCCATGTGGGCCAT-3′) with an annealing temperature of 59°C.

MicroRNA was isolated using mirVana miRNA Isolation Kit (Applied Biosystems, Waltham, MA, USA) and cDNA was synthesized using TaqMan MicroRNA Reverse Transcription Kit (Applied Biosystems). Real-time quantitative PCR for miR-200c was performed using TaqMan MicroRNA Assays according to the manufacturer's instructions (Applied Biosystems). miRNA expression levels were normalized to endogenous expression of U6 small nuclear RNA.

### Generation of stable cell lines

A fragment containing the coding sequence of myc-tagged TMPRSS4 from pCMV-myc-TMPRSS4 [16] was obtained by PCR with the following primer set (5′-TTTAAAGCCGCCATGGAGCAGAAA-3′ and 5′-TCTAGACTATTACAGCTCAGCCTTCCAG-3′) and then was subcloned into the pLVX-EF1α-IRES-Puro lentiviral vector (Clontech Laboratories, Inc., Mountain View, CA, USA), which was cleaved by EcoRI-XbaI (EcoRI cleavage was followed by the Klenow reaction to generate blunt ends before XbaI digestion), to generate pLVX-EF1α-IRES-Puro-TMPRSS4. For generation of lentiviruses, pLVX-EF1α-IRES-Puro-TMPRSS4 or the empty vector was co-transfected with the Lentiviral Packaging Mix (Sigma) into Lenti-X-293T cells (Clontech) using Lipofectamine 2000, and virus-containing supernatants were harvested and concentrated at 48 h post-transfection. PC3 cells were transduced with the lentiviruses for 12 h in the presence of polybrene (2 mg/ml) and were subsequently selected with puromycin (1 mg/ml) for 1 week to establish stable clones. The expression of myc-tagged TMPRSS4 was analyzed by immunoblotting.

### Mouse xenograft model

All animal procedures were performed in accordance with the guidelines of the Animal Care Committee at the Korea Research Institute of Bioscience and Biotechnology. Nude mice (BALB/c-nude, 5-week-old females) were obtained from Nara Biotech (Seoul, Korea). PC3 stable cells (vector transfectants and TMPRSS4-overexpressing cells) were injected subcutaneously into the right flank of each mouse (n = 3 or 4 per group). In brief, 5 × 10^6^ cells were resuspended in PBS and then mixed with Matrigel (BD Biosciences, San Jose, CA, USA) on ice before injection. Body weight and tumor volume were measured twice a week for 4 weeks. On day 28, mice were sacrificed and dissected tumor masses were photographed. The tumor volumes were calculated as follows: tumor volume = (**a** × **b**^2^) × 1/2, where **a** was the width at the widest point of the tumor and **b** was the maximal width perpendicular to **a**.

### Chromatin immunoprecipitation (ChIP)

ChIP assays were performed according to the instructions of the ChIP Assay Kit from Upstate Biotechnology (Lake Placid, NY, USA). Briefly, PC3 cells were co-transfected with a TMPRSS4 expression vector or an empty vector and siRNAs specific to c-Jun or ATF-2 for 48 h. The equivalent of 1 × 10^6^ transfected PC3 cells was used per ChIP reaction using rabbit anti-c-Jun or rabbit anti-ATF-2. As a control antibody, normal rabbit IgG was used. Immunoprecipitated and input DNA were analyzed by PCR with Slug promoter-specific primers (5′-GGCTCAGTTCGTAAAGGA-3′ and 5′-CATCTTGCCAGCGGGTCT-3′ for +1/+172).

### Promoter reporter assay

Cells were transfected with Lipofectamine 2000. For transfection, 2 × 10^5^ cells were seeded onto 6-well plates. After incubation for 24 h, 2 μg of reporter plasmid DNA and 1.8 μg of the TMPRSS4-, c-Jun-, or Slug-expressing vector were co-transfected. At 48 h post-transfection, firefly luciferase activity was measured using a Dual-luciferase reporter assay system (Promega, Southampton, UK). The transfection efficiency was normalized by measuring Renilla luciferase activity, encoded by the co-transfected Renilla luciferase vector (pRL-TK). The Slug promoter (−981/+174) construct in the pGL4-luciferase reporter vector was kindly provided by Dr. Shuang Huang (Medical College of Georgia, USA) [23]. The AP-1 cis-element reporter plasmid (AP-1 reporter) was purchased from Stratagene (La Jolla, CA, USA). The Cyclin D1 promoter (−962/+134) reporter plasmid was kindly provided by Addgene (#32727). Where indicated, cells were transiently transfected for 6 h and then treated with 20 mM PD98059, 15 mM SP600125, or 0.4% DMSO for 24 h before lysis.

### Invasion assay

Invasion assays were performed as described previously [16]. Cells were plated in serum-free medium on Transwell inserts (Corning, NY, USA) coated with 25 μg of Matrigel. The underside of the insert was pre-coated with 2 μg of collagen type I (Sigma). After incubation for 48 h at 37°C/5% CO_2_, inserts were fixed with 3.7% paraformaldehyde prepared in phosphate-buffered saline and stained with 2% crystal violet. The number of cells that had invaded was counted in five representative (×100) fields per insert.

### Analysis of the cancer genome atlas (TCGA) data

cBioPortal (www.cbioportal.org) [58, 59] was used to analyze TCGA-generated human prostate adenocarcinoma (MSKCC, Cancer Cell 2010) [21] and Cancer Cell Line Encyclopedia (CCLE)[31] data. All samples where mRNA expression profiles are available were included in our analysis. The Pearson's correlation coefficient (r) and *P*-value were calculated using the cBioPortal webpage and CGDS-R package (available at http://cran.r-project.org/web/packages/cgdsr/index.html). Survival curve analysis and Fisher's exact test were performed using the cBioPortal webpage tools.

### Statistical analysis

Statistical analyses were performed using the Student's *t*-test, Logrank test, Pearson test, and Fisher's exact test. *P* < 0.05 was considered statistically significant.

## SUPPLEMENTARY MATERIALS FIGURES



## Abbreviations

AP-1: activating protein-1
ATCC: American Type Culture Collection
ATF: activating transcription factor
CCLE: Cancer Cell Line Encyclopedia
EMT: epithelial-mesenchymal transition
ERK1/2: extracellular signal-regulated kinase 1/2
HEK293E: human embryonic kidney 293E
JNK: c-Jun N-terminal kinase
SD: standard deviation
siRNA: small interfering RNA
TCGA: The Cancer Genome Atlas
TTSP: type II transmembrane protease
uPA: urokinase-type plasminogen activator
