# Supplementary material for: TMPRSS4 induces invasion and proliferation of prostate cancer cells through induction of Slug and cyclin D1
Source: Oncotarget. 2016 Jul 2;7(31):50315–32. doi: 10.18632/oncotarget.10382 (PMC5226585; doi:10.18632/oncotarget.10382)
Supplement: Supplementary file 1 [file oncotarget-07-50315-s001.pdf]

TMPRSS4 induces invasion and proliferation of prostate cancer cells through induction of Slug and cyclin D1

SUPPLEMENTARY MATERIALS AND METHODS

Experiments

All experiments were performed as described in the Materials and Methods section.

SUPPLEMENTARY FIGURES

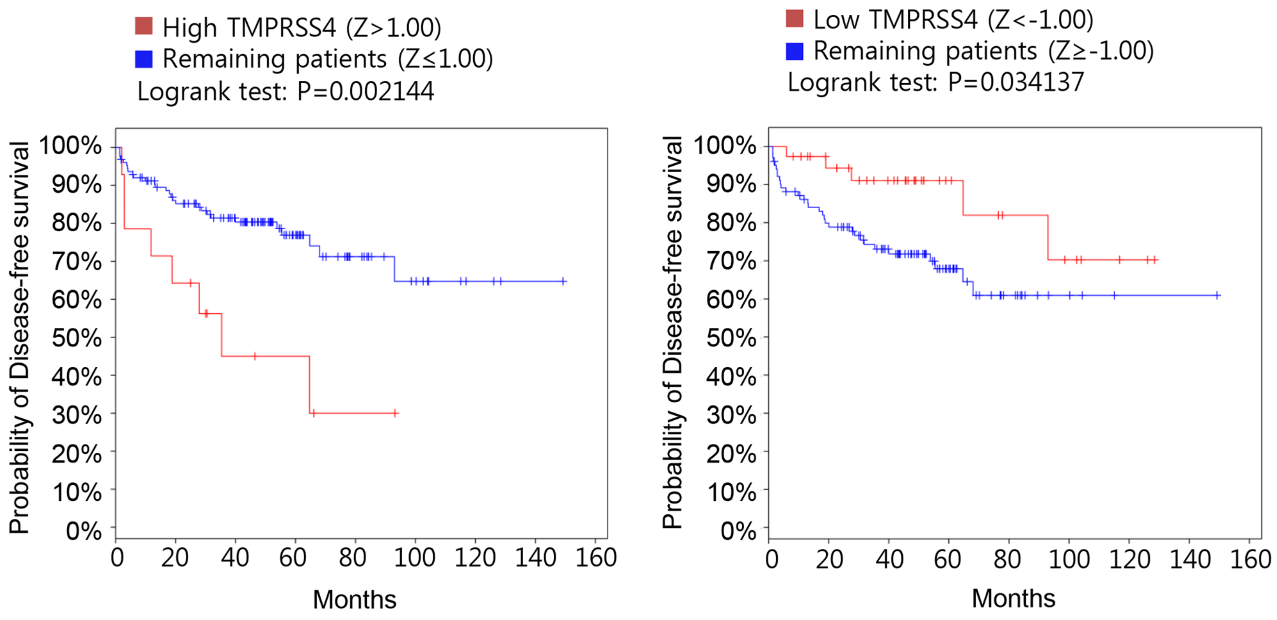

Supplementary Figure S1: TMPRSS4 expression correlated with the disease-free survival of prostate cancer patients. Kaplan-Meier analysis shows the probability of disease-free survival from prostate adenocarcinoma patient data (MSKCC, Cancer Cell 2010) in relation to TMPRSS4 mRNA expression. All tumors with an mRNA expression profile ($n = 150$) were analyzed. High TMPRSS4 expression is defined by $Z > 1.00$ (left) and low TMPRSS4 expression is defined by $Z < -1.00$ (right). *P* values were calculated by the Logrank test.

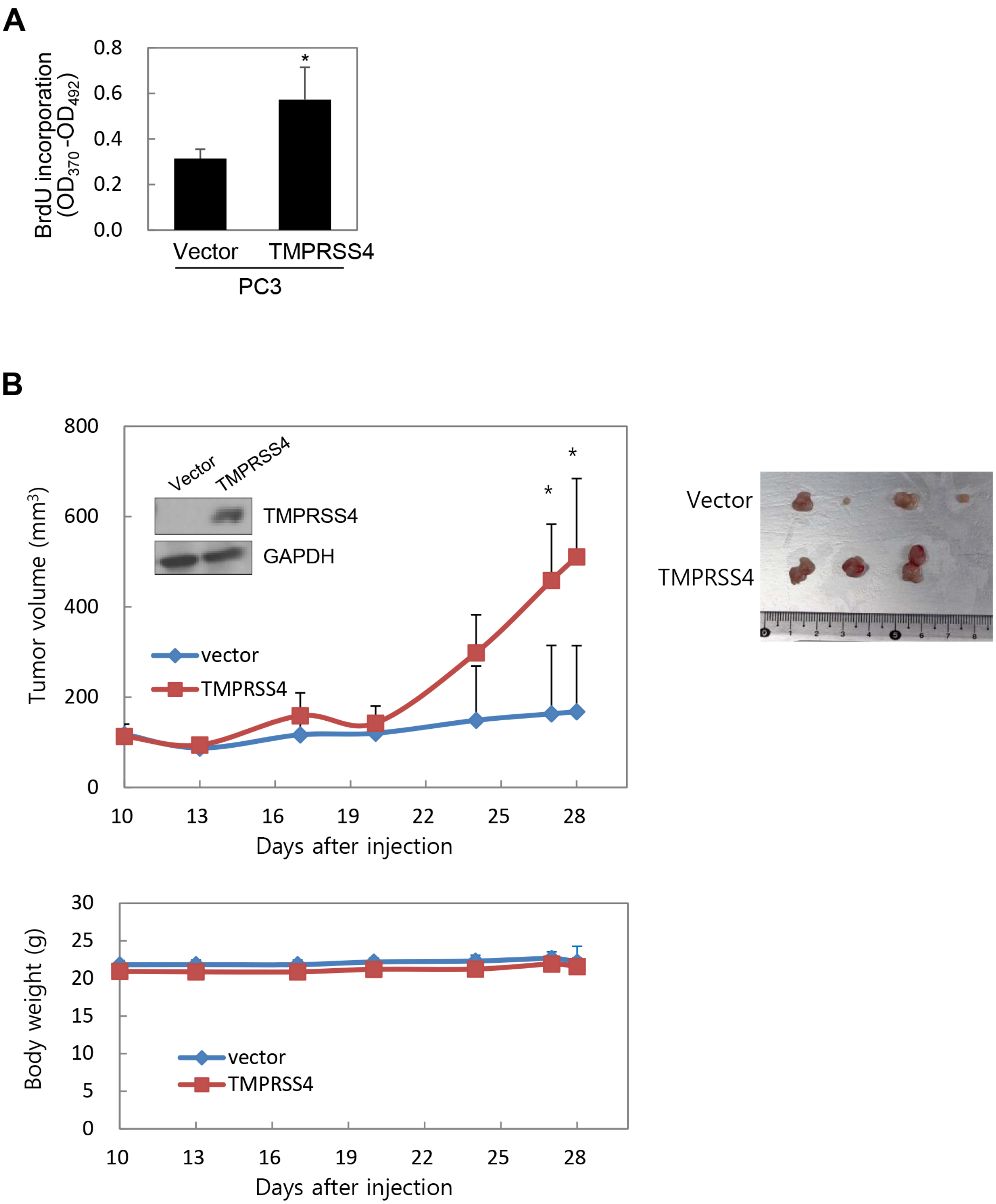

Supplementary Figure S2: A. TMPRSS4 enhanced S-phase progression of PC3 cells. PC3 cells were transfected with a TMPRSS4 expression vector for 48 h. Transfected cells were seeded onto 96-well plates at a density of 3000 cells/well and incubated for 48 h. Then, the cells were labeled with 10 mM BrdU for 2 h and the assay was performed as described in Materials and Methods. **B.** PC3 stable cells (vector transfectants and TMPRSS4-overexpressing cells) were generated as described in Materials and Methods. Expression of TMPRSS4 were determined by immunoblot analysis. PC3 stable cells were injected subcutaneously into the flank of nude mice (n = 3 or 4) as described in Materials and Methods. Body weight and tumor volume were measured for 4 weeks. Tumor volume was calculated using the formula, length × width^2/2. Values represent mean ± standard deviation (SD). * $P < 0.05$. (*Continued*)

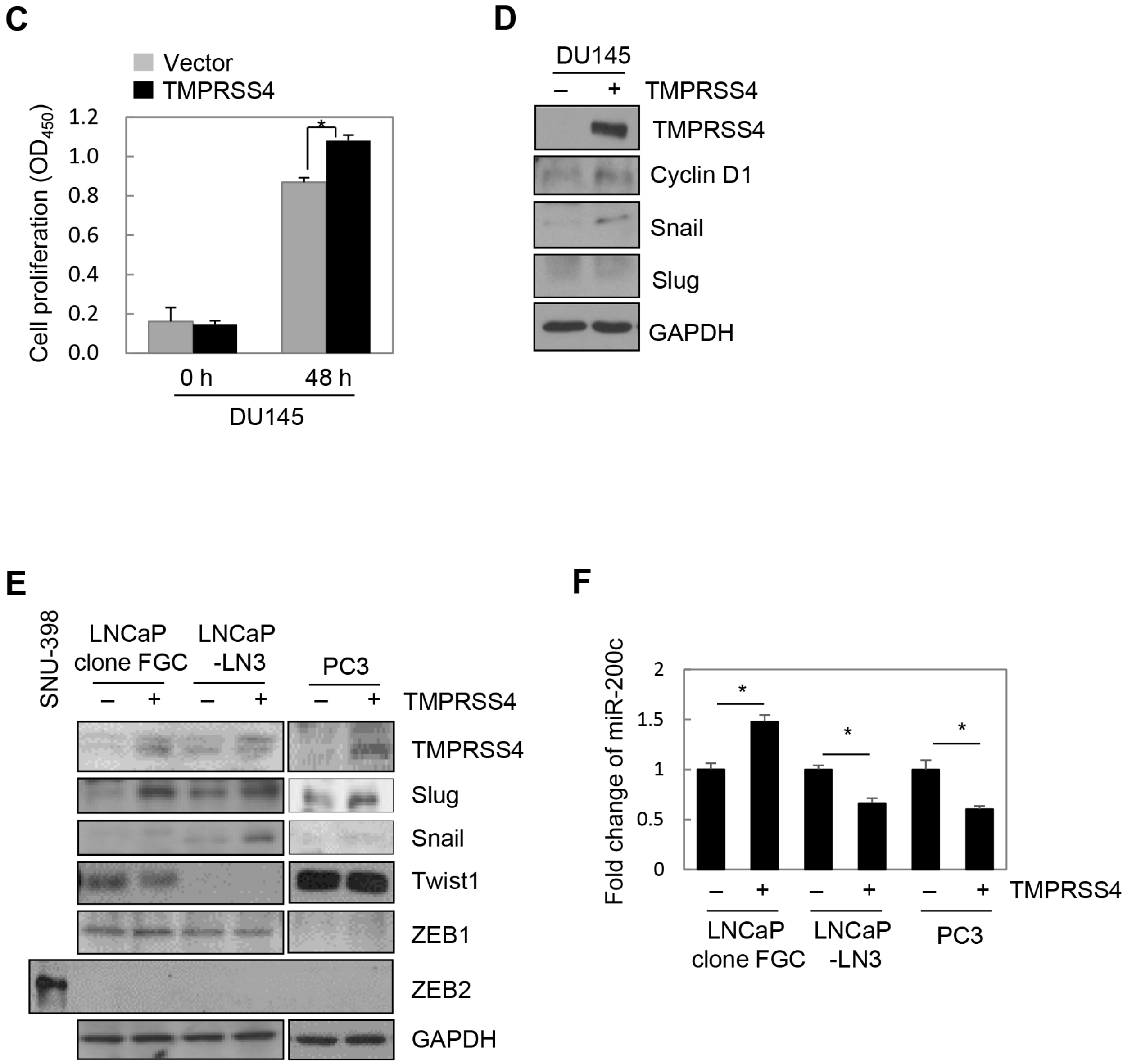

Supplementary Figure S2: (*Continued*) C. and **D.** TMPRSS4 induced proliferation and cyclin D1 expression in DU145 cells. DU145 cells were transfected with a TMPRSS4 expression vector for 48 h. (C) Transfected cells were seeded into 96-well plates at a density of 3000 cells/well and incubated for 48 h. Cell proliferation was determined by the colorimetric WST assay. Values represent mean ± SD. * $P < 0.05$. (D) Transfected cells were lysed and used for immunoblotting. An anti-myc antibody was used to detect myc-tagged TMPRSS4. GAPDH was used as an internal control. **E, F.** Cells were transfected with a TMPRSS4 expression vector for 48 h. (E) Transfected cells were lysed and used for immunoblotting. SNU-398 cell lysates were used as a positive control for detecting ZEB2. (F) MicroRNA was isolated and cDNA was synthesized as described in Materials and Methods. Real-time quantitative PCR for miR-200c was performed.

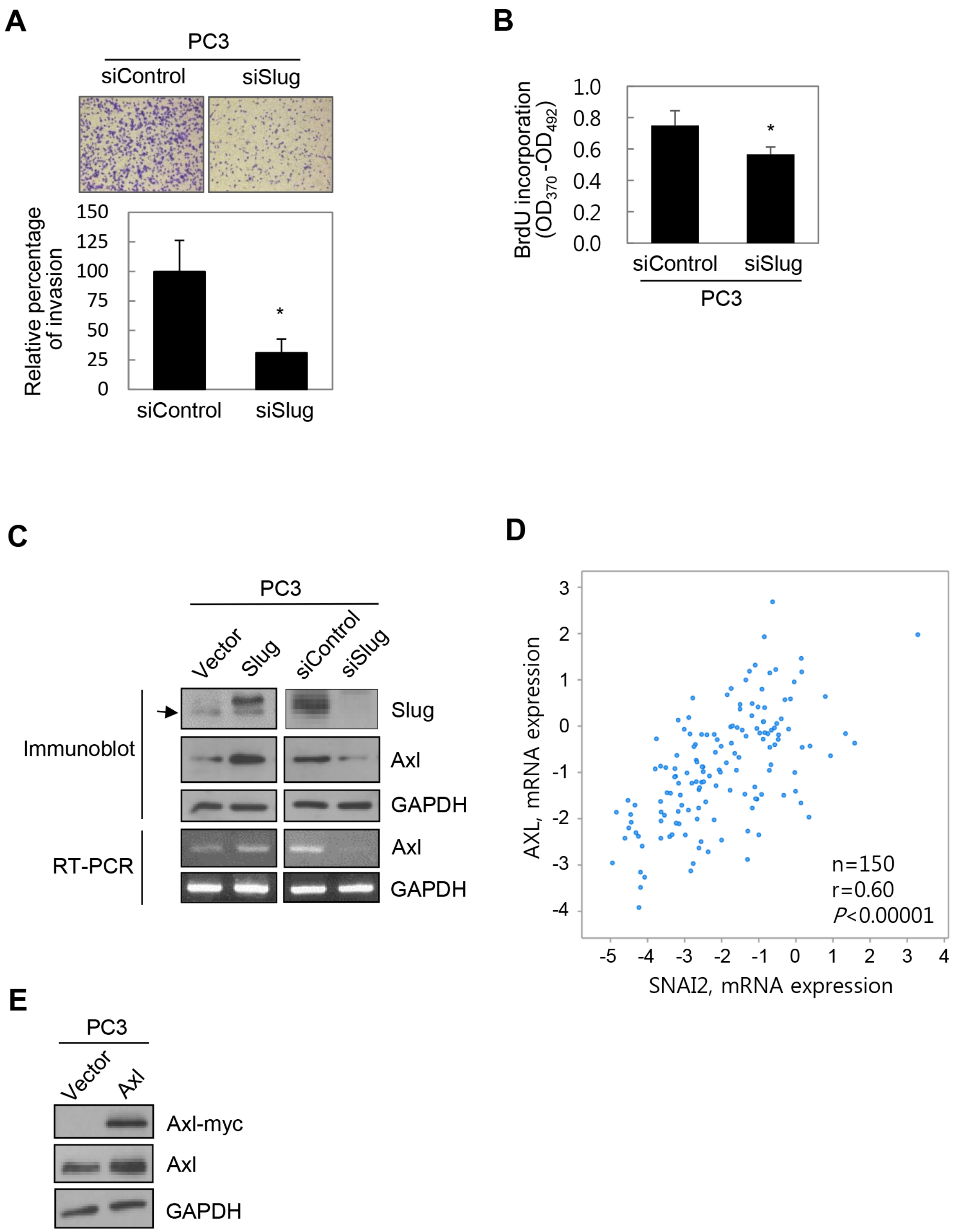

Supplementary Figure S3: A, B. PC3 cells were transfected with siRNA specific to Slug for 48 h. (A) Transfected cells were allowed to invade Matrigel (1×10^4 cells) for 48 h. The number of cells that had invaded was counted in five representative (×100) fields per Transwell insert. (B) Transfected cells were seeded onto 96-well plates at a density of 3000 cells/well and incubated for 48 h. Then, the cells were labeled with 10 mM BrdU for 6 h and the assay was performed as described in Materials and Methods. Values represent mean ± SD. * $P < 0.05$. **C.** PC3 cells were transfected with a Slug expression vector or siRNA specific to Slug for 48 h. Transfected cells were lysed for immunoblotting and RT-PCR analysis. Arrow indicates endogenous Slug. **D.** Scatter plots examining Slug mRNA expression (x-axis) and Axl mRNA expression (y-axis) from prostate adenocarcinoma data (MSKCC, Cancer Cell 2010). Primary (n = 131) and metastatic (n = 19) tumors with an mRNA expression profile were included. Correlation was statistically analyzed using the Pearson test. **E.** PC3 cells were transfected with an Axl expression vector for 48 h before lysate preparation for immunoblotting. Anti-myc and anti-Axl antibodies were used to detect exogenous myc-tagged Axl and total Axl, respectively.

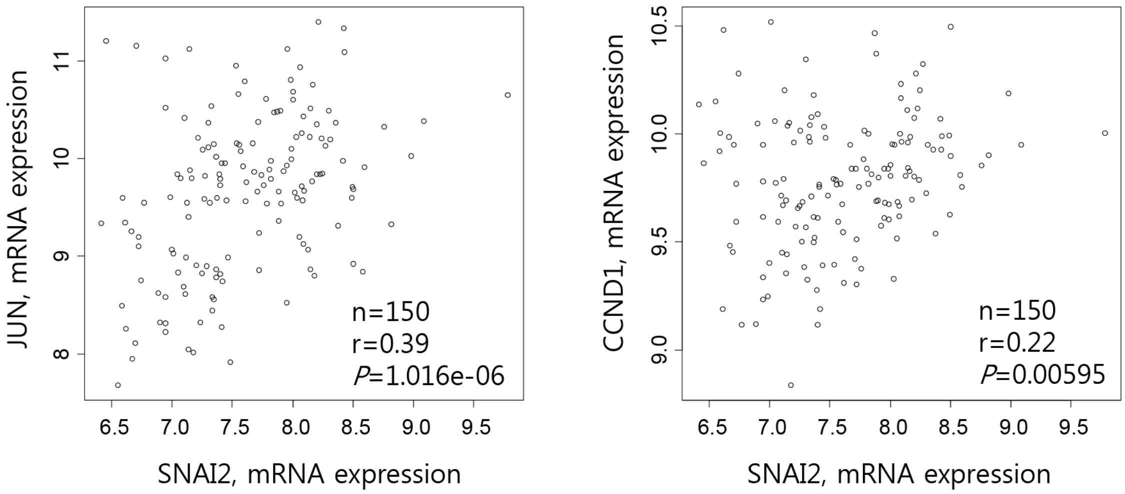

Supplementary Figure S4: Scatter plots examining Slug mRNA expression (x-axis) and c-Jun (left) or cyclin D1 (right) mRNA expression (y-axis) from prostate adenocarcinoma data (MSKCC, cancer cell 2010). Primary (n = 131) and metastatic (n = 19) tumors with an mRNA expression profile were included. Correlations were statistically analyzed using the Pearson test.

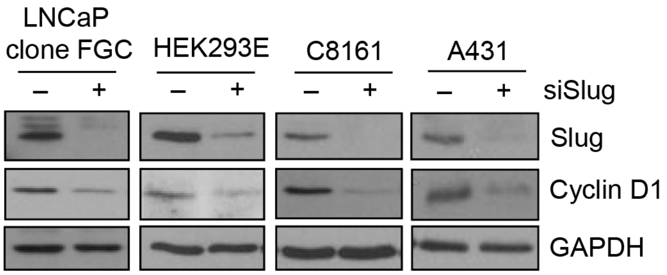

Supplementary Figure S5: Cells were transfected with siRNA specific to Slug for 48 h. Transfected cells were lysed and used for immunoblotting. GAPDH was used as an internal control.
